# miR-494-3p Regulates Cellular Proliferation, Invasion, Migration, and Apoptosis by PTEN/AKT Signaling in Human Glioblastoma Cells

**DOI:** 10.1007/s10571-015-0163-0

**Published:** 2015-02-08

**Authors:** Xue-tao Li, Hang-zhou Wang, Zhi-wu Wu, Tian-quan Yang, Zhao-hui Zhao, Gui-lin Chen, Xue-shun Xie, Bin Li, Yong-xin Wei, Yu-lun Huang, You-xin Zhou, Zi-wei Du

**Affiliations:** 1 Department of Neurosurgery & Brain and Nerve Research Laboratory, The First Affiliated Hospital of Soochow University, 188 Shizi Street, Suzhou, 215006 Jiangsu China; 2 Department of Neurosurgery, Children’s Hospital Affiliated to Soochow University, 303 Jingde Street, Suzhou, 215006 Jiangsu China

**Keywords:** Glioma, MicroRNA, Migration, Apoptosis, PTEN

## Abstract

Malignant gliomas are the most common primary brain tumors, and the molecular mechanisms involving their progression and recurrence are still largely unclear. Substantial data indicate that the oncogene miR-494-3p is significantly elevated in gliomas, but the molecular functions of miR-494-3p in gliomagenesis are largely unknown. The present study aimed to explore the role of miR-494-3p and its molecular mechanism in human brain gliomas, malignant glioma cell lines, and cancer stem-like cells. The expression level of miR-494-3p in 48 human glioma issues and 8 normal brain tissues was determined using stem-loop real-time polymerase chain reaction (PCR). To study the function of miR-494-3p inhibitor in glioma cells, the miR-494-3p inhibitor lentivirus was used to transfect glioma cells. Transwell invasion system was used to estimate the effects of miR-494-3p inhibitor on the invasiveness of glioma cells. A mouse model was used to test the effect of miR-494-3p inhibitor on glioma proliferation and invasion in vivo. Results showed that the expression of miR-494-3p in human brain glioma tissues was higher than in normal brain tissues. Downregulated expression of miR-494-3p can inhibit the invasion and proliferation and promote apoptosis in glioma cells. Quantitative reverse transcription PCR and Western blotting analysis revealed that the expression of PTEN was increased after downexpression of miR-494-3p in glioma cells (U87 and U251). miR-494-3p inhibitor could prevent migration, invasion, proliferation, and promote apotosis in gliomas through PTEN/AKT pathway. Therefore, the study results have shown that miR-494-3p may act as a therapeutic target in gliomas.

## Introduction

Glioblastoma is the most common primary brain tumor, accounting for nearly 40 % of all central nervous system malignancies (Frank et al. 2007). They have the ability of higher invasion, migration, proliferation, and lower apoptosis. In spite of advances in surgery, radiation therapy, and chemotherapy, the prognosis of patients with gliomas has not significantly improved and the median survival time of patients is expected to be 12–15 months (Wen and Kesari 2008). The poor prognosis of gliomas is largely attributed to their rapid growth, invasive/migratory nature, and high rate of recurrence. Therefore, a novel therapeutic strategy would involve suppression of proliferation, inhibition of glioma cell migration, and promotion of glioma cell apoptosis.

Phosphatase and tensin homolog, which is deleted on chromosome 10 (PTEN), is one of the most frequently lost tumor suppressors in various types of cancers in humans (Dimpy et al. 2006). Over the past decades, some researchers have found that the PTEN gene plays the most important role in the progression of gliomas (Kim et al. 2011). In agreement with their antioncogenetic role, PTEN mediates cell proliferation, migration, apoptosis, and angiogenesis by activating the AKT pathway (Davies et al. 1998a, b; Mehrian et al. 2007). PTEN has often been reported to be abnormally expressed in breast, liver, lung, and brain cancers (Estrella et al. 2014; Xu et al. 2014). Some researchers have found that PTEN messenger ribonucleic acid (mRNA) can be regulated by microRNA (miRNA).

miRNAs are small, endogenous, noncoding RNAs that regulate gene expression by antisense complementarity to specific mRNAs. Cumulative evidences indicate that miRNAs regulate diverse biologic processes such as cell migration, invasion, proliferation, identity, apoptosis, stress resistance, and stem cell maintenance (Gabriely et al. 2008; Hwang and Mendell 2006). miRNAs can target different genes including oncogenes and antioncogenes, while some miRNAs function as tumor suppressors and others act as oncogenes in various cancers (Yang et al. 2012; Li et al. 2012). In the past decades, many microRNAs such as miR-21, miR-155, miR-16, and miR-218 have been found to regulate the progress of gliomas (Yang et al. 2014a, b; Yanyang et al. 2013; Zhou et al. 2013). However, the function of miR-494-3p in regulating glioma progression is poorly understood.

The present study aimed to explore the role of miR-494-3p and its molecular mechanism in human brain gliomas, malignant glioma cell lines, and cancer stem-like cells. miR-494-3p was significantly upregulated in glioma cells and clinical glioma tissues compared to normal brain tissues. It could be assumed that miR-494-3p can promote glioma cell invasion and proliferation and inhibit its apoptosis. Downexpression of miR-494-3p in glioma cells and their stem cell-like cells markedly suppresses the motility, invasion, and proliferation; and it promotes their apoptosis. miR-494-3p can target the PTEN mRNA. Therefore, miR-494-3p may inhibit the expression of PTEN protein and promote the progression of gliomas.

## Materials and Methods

### Patients and Samples

Forty-three glioma samples were prospectively obtained from 29 Chinese patients between March 2009 and September 2013 at the Department of Neurosurgery, Brain, and Nerve Research Laboratory of the First Affiliated Hospital of Soochow University. Forty-three patients with glioma (28 men and 15 women) were also included. Their glioma condition was graded based on the 2007 World Health Organization (WHO) Classification System. Eight normal brain tissue samples were obtained from adult patients with craniocerebral injuries; for whom, a partial resection of brain tissue was required as decompression treatment to reduce intracranial pressure. All human samples were used in accordance with the policies of the Institutional Review Board of the First Affiliated Hospital of Soochow University.

### Cell Lines and Cancer Stem-Like Cell Culture

U87MG and U251MG were purchased from the Chinese Academy of Sciences Cell Bank in 2012. They were grown in Dulbecco’s modified Eagle’s medium (DMEM; Hycolne, Thermo Fisher Scientific, Waltham, MA, USA) supplemented with 10 % fetal bovine serum (FBS; Gibco) and 100 U/mL penicillin/streptomycin (Gibco).

### Quantitative Real-Time Polymerase Chain Reaction (qRT-PCR) Analysis of mRNA and miRNA Expression

Total RNA from tissues and cells was isolated using TRIzol reagent (Invitrogen) for both mRNA and miRNA analyses. The expression level of miR-494-3p was carried out using All-in-One miRNA qRT-PCR Detection Kit (GeneCopoeia) per manufacturer’s instructions (Applied LightCycler480) and was normalized to the expression of RNU6. The expression of PTEN mRNA was determined using SYBR Green I qRT-PCR Kit per manufacturer’s instructions (Applied LightCycler480) and was normalized to the expression of glyceraldehyde 3-phosphate dehydrogenase mRNA. The following primers were used: PTEN mRNA forward: 5′-AAGCTGGAAAGGGACGAACT-3′, reverse: 5′-ACACATAGCGCCTCTGACTG-3′ and GAPDH mRNA forward: 5′-CCTCTCTCTAATCAGCCCTCTG-3′, reverse: 5′-AGAAGGCTGGGGCTCATTTG-3′ (Sangon, Shanghai, China).

### In Vitro Migration and Invasion Assays

U87 and U251 were transfected with miR-494-3p inhibitor or negative control and kept in a 6-well insert. After cultured for 24 h, the transfected cells were transferred to the top of the matrigel-coated invasion chambers (24-well insert, 8um pore size, BD) in serum-free DMEM. To the lower chambers, DMEM contain 10 % FBS were added as a chemoattractant. In the cultured system, noninvading cells from the top wells were removed by a cotton wrap. Cells on the lower membrane surface were fixed in 4 % formaldehyde and stained with 0.2 % crystal violet. Invading cells were manually counted in five randomly chosen fields under a microscope, and photographs were used.

### 3-(4,5-Dimethylthiazol-2-yl)-2,5-Diphenyltetrazolium Bromide (MTT) Assay

Twenty-four hours after transfection with miR-494-3p and negative control, 1,000 cells in 150 µL media per well were seeded and cultured in a 96-well plate for 24 h. MTT was diluted to 5 mg/mL in phosphate buffered saline (PBS) and 20 µL were added to each well. After 4 h, the cells were resuspended in 200 µL of dimethyl sulfoxide (DMSO) and shaken for 15 min. The absorbance was measured at 570 nm, with 655 nm as the reference wavelength. All experiments were performed in triplicate.

### Analysis of Apoptosis

The apoptosis of the cells infected with miR-494-3p inhibitor vector or negative control vector was assayed by staining with Annexin V-PE (BD Biosciences, USA) and detected by flow cytometry. Cells were collected and washed with ice-cold PBS 5 days after transfection and stained with 100 µL binding buffer containing 5 µL Annexin V-PE at room temperature in the dark for 10–15 min. For each experiment, 20,000 cells were analyzed using the FACSCalibur flow cytometer. All the experiments were performed in triplicate.

### Western Blot Analysis

Forty-eight hours after miR-494-3p inhibitor and negative control transfection, total protein lysate was extracted using radioimmunoprecipitation assay lysis buffer (50 mM Tris/HCl pH 7.5, 0.1 % sodium dodecyl sulfate (SDS), 1 % Triton-X 100, 0.5–1 % sodium deoxycholate, 150 mM sodium chloride, and protease inhibitors (Roche). Protein samples were separated with 10 % SDS–polyacrylamide gel electrophoresis (SDS-PAGE) and transferred onto nitrocellulose membranes. Membranes were incubated with primary antibodies overnight at 4 °C. Membranes were washed and incubated for 2 h with horseradish peroxidase (HRP)-conjugated anti-rabbit secondary antibodies (Prosci Inc. 1:3,000; Poway, CA, USA), followed by detection and visualization using ECL Western blotting detection reagents (Pierce antibodies, Thermo Fisher,USA). The primary antibodies used were anti-PTEN (1:1,000; Abcam, Tokyo, Japan), anti-BCL2 (1:500; Abcam, Tokyo, Japan), anti-Bax (1:1,000; Abcam, Tokyo, Japan), anti-matrix metallopeptidase (MMP)-2 (1:500; Abcam, Tokyo, Japan), and anti-MMP9 (1:1,000; Abcam, Tokyo, Japan), anti-caspase3, and caspase9 (1:1,000; Abcam, Tokyo, Japan).

### Xenografted Tumor Model

Computer-designed oligonucleotide sequences expressing the miR-494-3p inhibitor were connected to the EmGFP-miR lentivirus. The lentivirus was transfected with U87 cells using transfection enhanced buffer. To select the stably transfected U87 cells, cells with efficiency about 80–90 % were transfected and cultured for 1 month and subcultured for more than 10 generations. Furthermore, the transfected cells (1 × 10^5^) were implanted in the flanks of athymic mice to establish the intracranial tumor models. All mouse experiments were carried out in accordance with institutional guidelines and regulations of the government.

### Immunohistochemistry and Immunofluorescence

Glioma tissue paraffin specimens and U87 glioma cell transplantation tumor specimens were used only in the laboratory. The specimens were cut with a microtome in 4 µm sections. Antigen retrieval was performed in 10 mM sodium citrate buffer of pH 6 for 10 min at 100 °C. Slides were incubated with primary antibodies against MMP9, Ki-67 (Boster Bioengineering Co., Wuhan, China), and PTEN at 4 °C for night. Sections were subsequently incubated with the Cell and Tissue Staining Kit HRP-3,3′-diaminobenzidine system (R&D Systems, Minneapolis, MN, USA), per the manufacturer’s instructions. Immunostainings were run with known positive and negative tumor controls.

### Statistical Analysis

Statistical analyses were performed using SPSS version 13.0 (SPSS, Chicago, IL, USA), and significance was determined with two-tailed Student’s *t* test. Data were considered statistically significant at *p* < 0.05.

## Result

### Baseline Characteristics

The mean ages of the patients at the time of surgery were 49.6 years for men and 48.2 years for women. There were 2 cases of grade I (pilocytic astrocytoma), 10 cases of grade II (diffuse astrocytoma), 10 cases of grade III (anaplastic astrocytoma), and 13 cases of grade IV (primary brain glioblastoma), according to the 2007 WHO’s classification system.

### Expression of miR-494-3p and PTEN mRNA were Inversely Correlated in Glioma Tissues and Cells

**Fig. 1:**
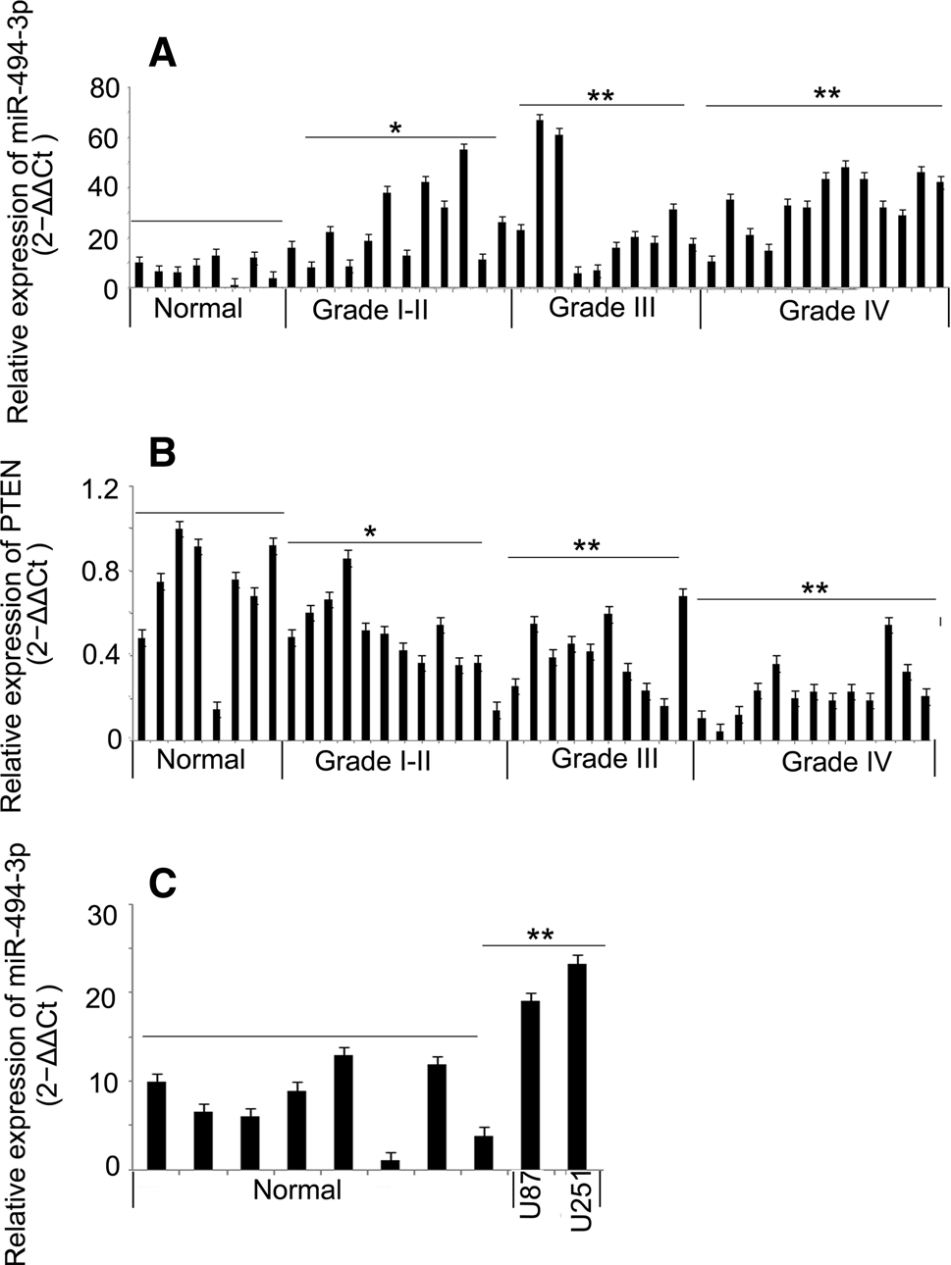
Expression of miR-494-3p and PTEN mRNA were inversely correlation in glioma tissues and cells. **a**, **b** The expression of miR-494-3p in glioma tissues and glioma cell lines were higher than in normal brain tissues (*p* < 0.05). **c** The expression of PTEN mRNA in glioma tissues was lower than in normal brain tissues (*p* < 0.05)

The expression level of miR-494-3p in 35 human glioma issues and 8 normal brain tissues was determined using stem-loop real-time PCR systems. Compared with control and normal brain tissues, the expression level of miR-494-3p was significantly upregulated in glioma tissues (Fig. 1a, b). Based on the available literature that the expression of miR-494-3p might be target the PTEN mRNA and could suppress the expression of PTEN mRNA, the expression of the gene in 35 human glioma tissues and 8 normal brain tissues was detected. In this study, the expression of PTEN mRNA was significantly downregulated in glioma tissues, and the expression of PTEN and miR-494-3P was inversely correlated in glioma tissues (Fig. 1c).

### miR-494-3p Inhibitor Lentivirus-Transfected Glioma Cell and miR-494-3p Inhibitor can Suppress the Proliferation of Glioma Cells

**Fig. 2:**
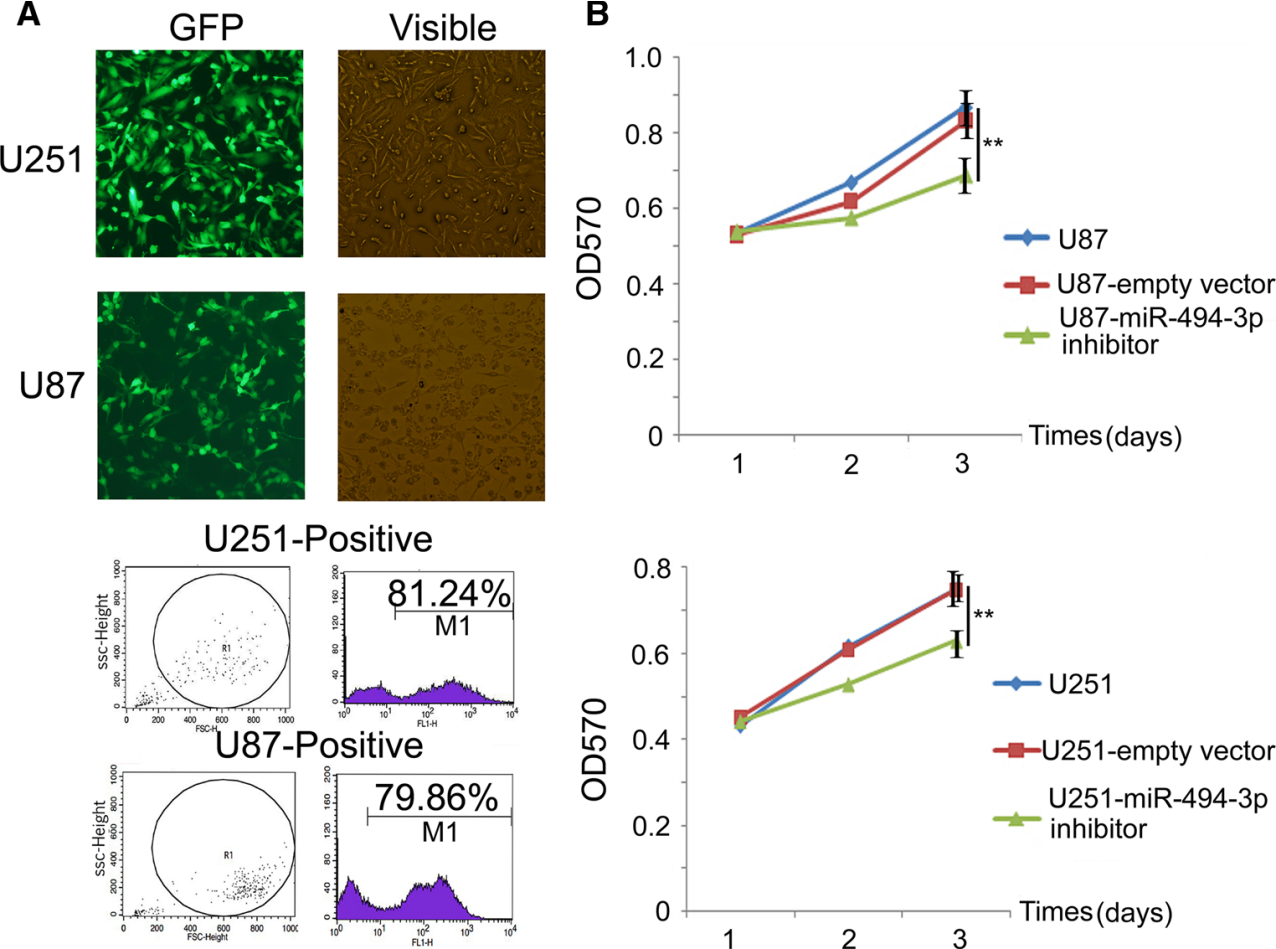
miR-494-3p inhibitor could suppress the proliferation of glioma cells. **a** Establishment of glioblastoma stable cell lines (magnification, ×400) and transfection efficiency (flow cytometry). **b** MiR-494-3p inhibitor suppress the proliferation of glioblastoma cells (U87 and U251) in vitro measured by MTT assay (**p < 0.01)

To study the function of miR-494-3p inhibitor in glioma cells, the miR-494-3p inhibitor lentivirus was used to transfect glioma cells. The transfection efficiency remained more than 75 % (Fig. 2a). A MTT proliferation assay demonstrated that cell proliferation was decreased in U87 and U251 glioma cells that were transfected with the miR-494-3p inhibitor vector compared with the empty vector cells or untreated cells (Fig. 2b).

### miR-494-3p Inhibitor Reduces Invasiveness of Glioma Cells In Vitro

**Fig. 3:**
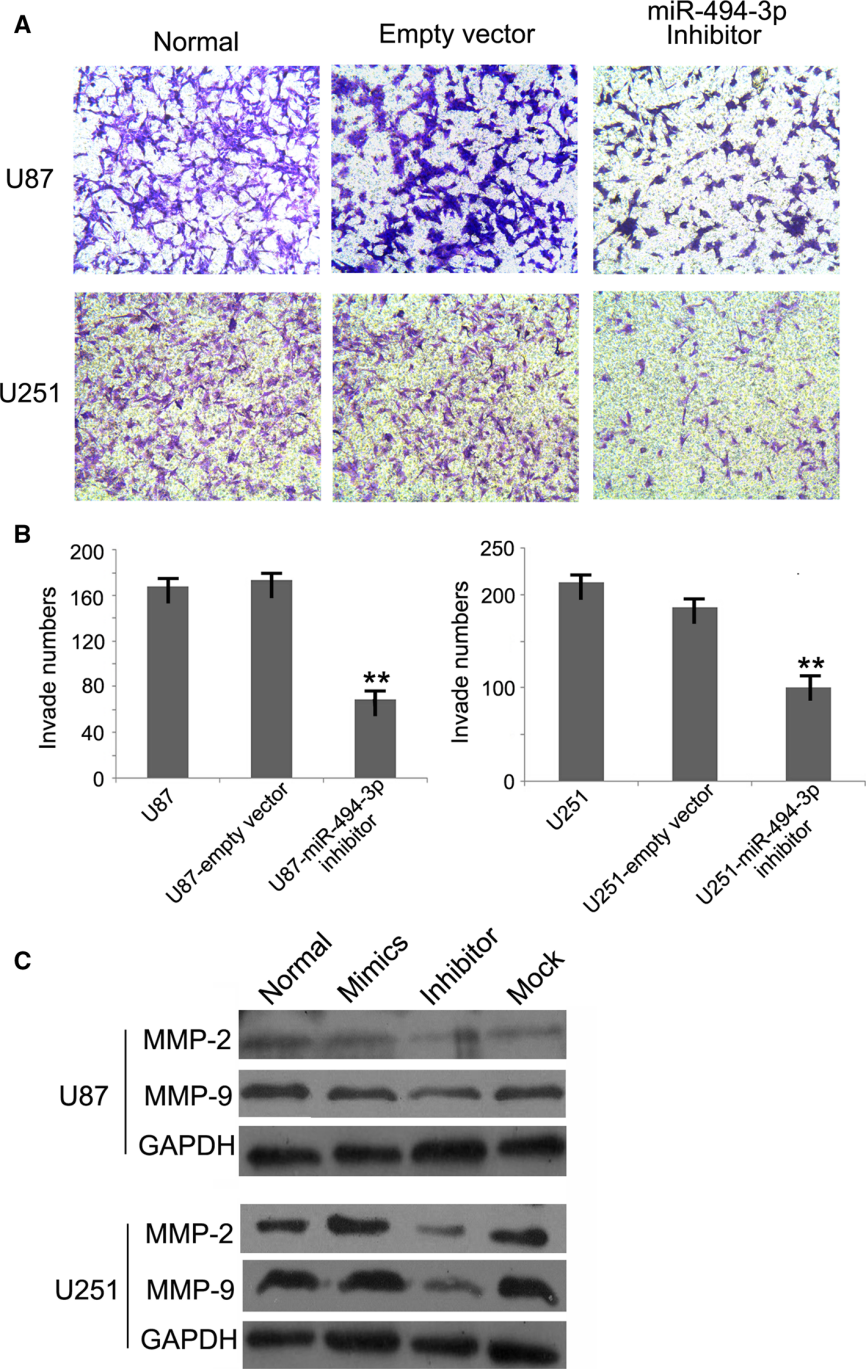
miR-494-3p inhibitor could reduce invasiveness of glioma cells in vitro. **a**, **b** The numbers of invasive cells with miR-494-3p inhibitor vector were significantly reduced compared with the empty vector cells or untreated cells (**p < 0.01). **c** The protein expression of MMP9 and MMP2 was obviously reduced by miR-494-3p inhibitor

Transwell invasion system was used to estimate the effects of miR-494-3p inhibitor on the invasiveness of glioma cells. The numbers of invasive cells with miR-494-3p inhibitor vector were significantly reduced compared with the empty vector cells or untreated cells (Fig. 3a, b). To further study the mechanism of the effects of miR-494-3p, Western blot was used to measure the proteins MMP2 and MMP9 (Fig. 3c).

### miR-494-3p Inhibitor Promotes the Apoptosis in Human Glioma Cells

**Fig. 4:**
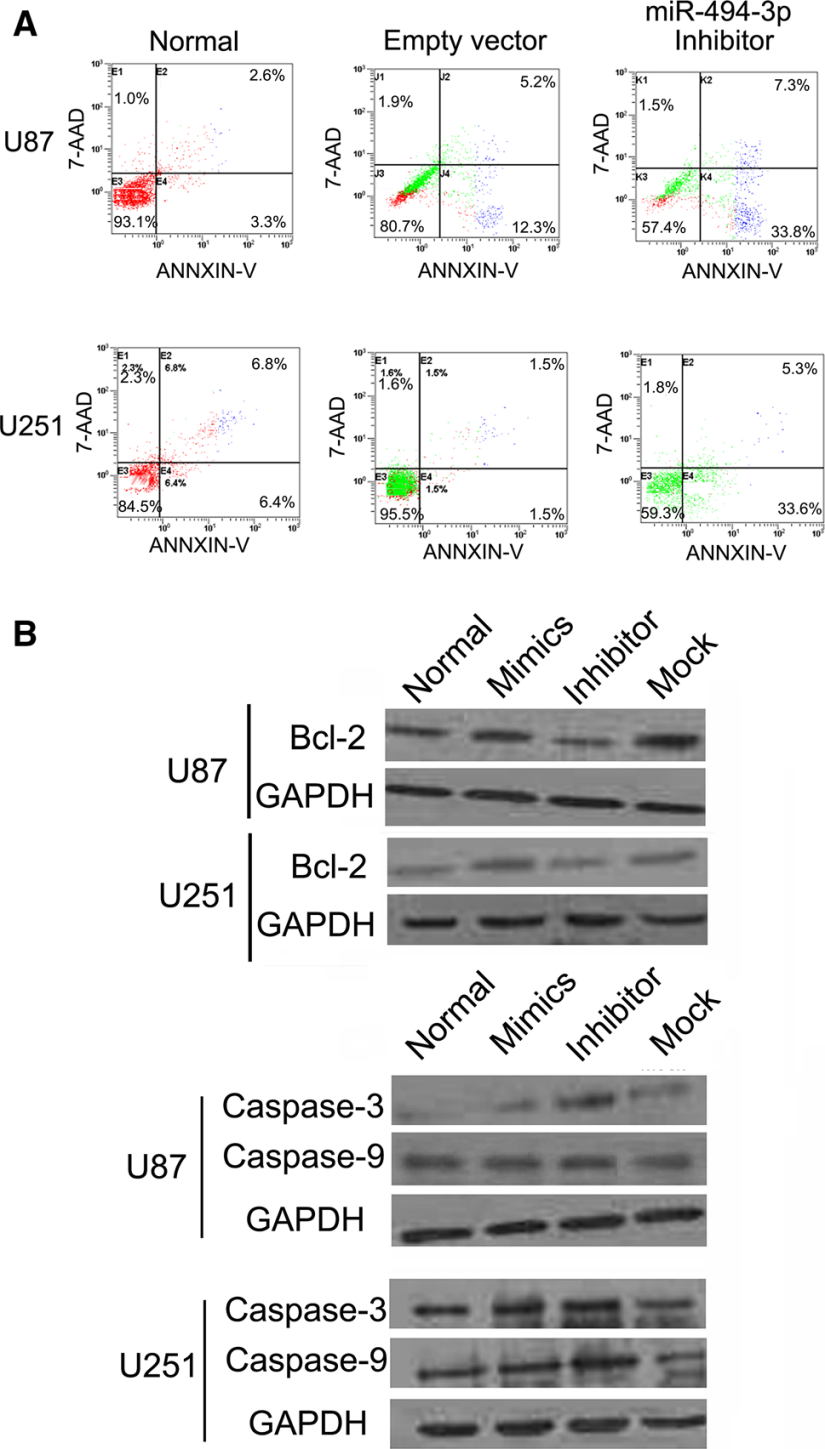
miR-494-3p inhibitor could promote the apoptosis in human glioma cells. **a** miR-494-3p inhibitor induced early apoptosis, compared with the empty vector cells or untreated cells and that the proportion of early apoptotic cells in the miR-494-3p inhibitor treatment group was markedly increased (*p* < 0.05, *n* = 3). **b** miR-494-3p inhibitor led to significant downregulation of BCL2 expression and upregulation of Caspase-3 and Caspase-9 expression

It was examined whether miR-494-3p inhibitor could facilitate apoptosis in human glioma cells. Flow cytometry data indicated that decreasing miR-494-3p expression could induce early apoptosis, compared with the empty vector cells or untreated cells and that the proportion of early apoptotic cells in the miR-494-3p inhibitor treatment group was markedly increased (*p* < 0.01, *n* = 3) (Fig. 4a). The mechanism of transfection of the miR-494-3P inhibitors on inducing apoptosis in human glioma cells was further investigated. BCL2, Caspase-3, and Caspase-9 were detected by Western blot. Data showed that the downregulation of miR-494-3p led to significant downregulation of BCL2 expression and upregulation of Caspase-3 and Caspase-9 expressions (Fig. 4b).

### miR-494-3P Inhibitor Reduces Cell Proliferation and Promote Cell Apoptosis Through PTEN/AKT Signaling

**Fig. 5:**
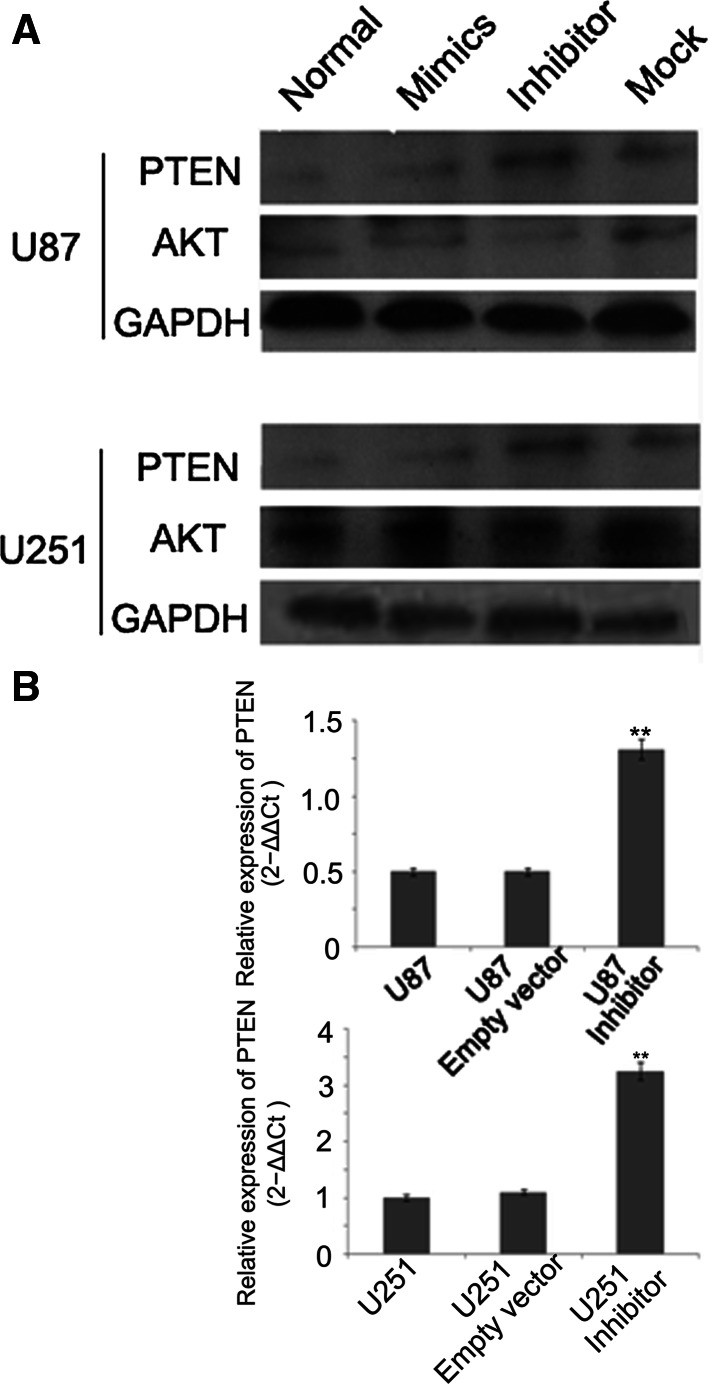
miR-494-3P inhibitor reduced cell proliferation and promoted cell apoptosis through PTEN/AKT signaling. **a** miR-494-3p inhibitor increased the protein expression of PTEN and decreased the protein expression of AKT. **b** miR-494-3p inhibitor upregulated PTEN mRNA in glioma cells (**p < 0.01)

miRNAs influence biological functions by inversely regulating their target genes. PTEN is a direct target of miR-494-3p in HEK-293 cell. The protein of PTEN was measured by Western blot. The expression of PTEN was upregulated in the group transfected with miR-494-3p inhibitor vector compared to the empty vector cells or untreated cells (Fig. 5a). The increase in PTEN expression or activity could contribute to the decreased AKT activation and led to subsequent growth, invasion, and survival in many cell types. miR-494-3p downexpression led to decreased AKT phosphorylation in glioma cells (Fig. 5a). In addition, the immunofluorescence method and Q-PCR systems were used to detect the expression of PTEN in the glioma cells. Upexpression of PTEN in transfected miR-494-3p inhibitor vector was observed compared to the empty vector cells or untreated cells (Fig. 5b).

### Downregulation of miR-494-3p Reduced Glioma Growth and Invasiveness in Glioma Xenograft Model

**Fig. 6:**
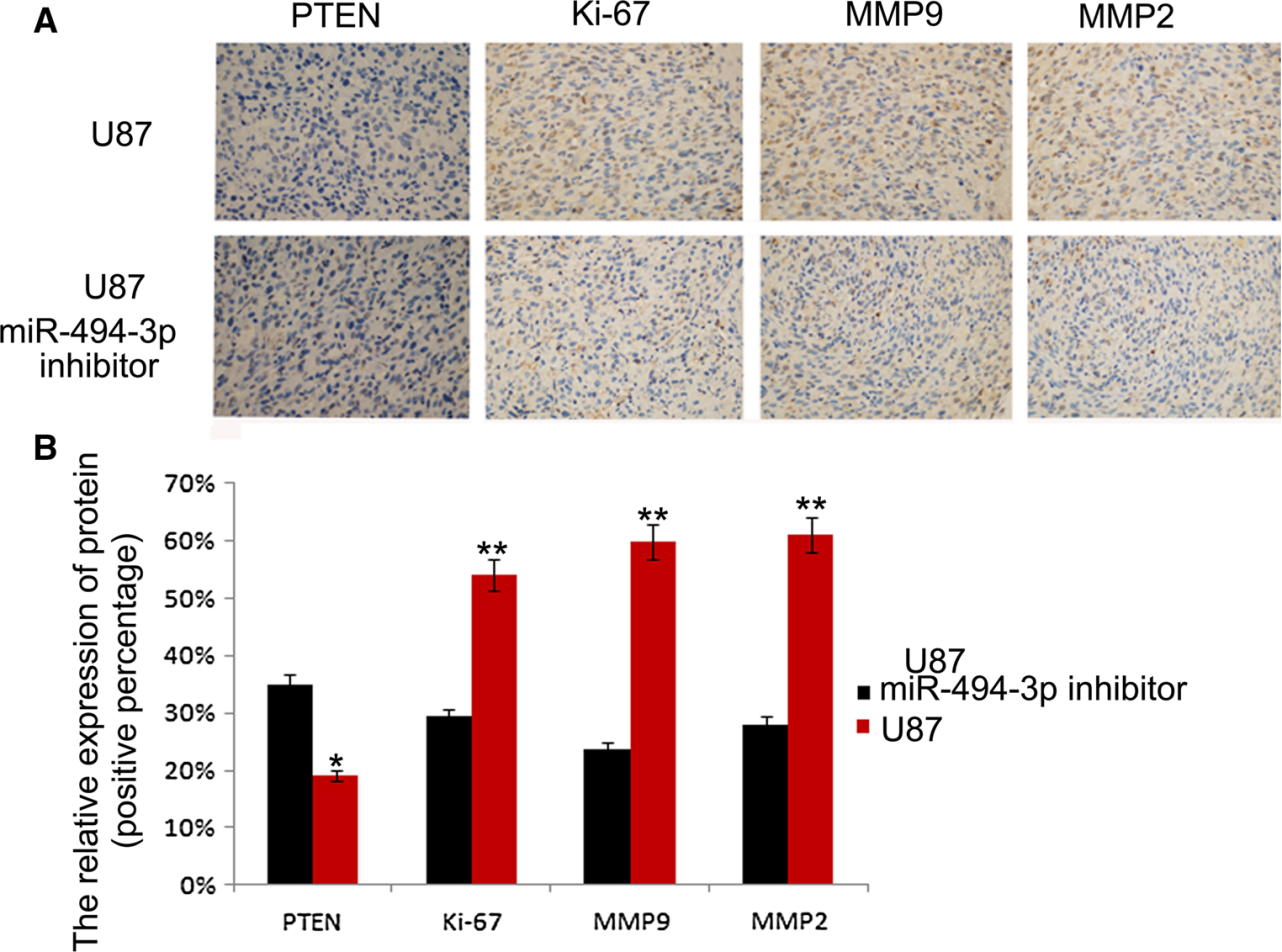
miR-494-3p can inhibit the cell proliferation in xenografted tumor model. The expression of Ki-67, PTEN, MMP2, and MMP9 in vivo (×400)

A mouse model was used to test the effect of miR-494-3p inhibitor on glioma proliferation and invasion in vivo. To further study the function, immunohistochemical method was used to detect the expression of Ki-67, PTEN, MMP2, and MMP9. We measured the relative expression of Ki-67, PTEN, MMP2, and MMP9 by percentage of positive cells; we found that the expression of the protein in U87-miR-494-3p inhibitor were changed compared to U87 (*p < 0.05, **p < 0.01) (Fig. 6).

Above all, downregulation of miR-494-3p could reduce glioma proliferation and invasion and promote apoptosis.

## Discussion

The present study explored the role of miR-494-3p and its molecular mechanism in human brain gliomas and malignant glioma cell lines. Previous studies showed that miRNA could act as a cancer gene, and tumor-suppressor genes might play a role in the occurrence and development of glioma (Van Wynsberghe et al. 2011; Reifenberger and Collins 2004; Croce et al. 2005). For instance, miR-16 and miR-218 were proved to be acted as cancer genes (Yang et al. 2014a, b; Tu et al. 2013). miR-21 and miR-211 were proved to be acted as tumor-suppressor genes (Yu Ren et al. 2010; Qiang et al. 2014). In the present study, miR-494-3p was found to be act as a cancer gene that could promote glioma cell proliferation through the downexpression of PTEN, a tumor-suppressor gene (Kwak et al. 2014; Liu et al. 2012). Furthermore, the function of miR-494-3p in glioma cells could be activated through the signaling pathway of PTEN/AKT.

miR-494, a well-known miRNA, is most frequently upexpressed in several different types of human cancers; and it has been found to be implicated in multiple cell processes including cell proliferation, apoptosis, and invasion. miR-494 acts as an antioncogene in gastric carcinoma by targeting c-myc (Li et al. 2014; Shen et al. 2014). Another study has shown that miR-494 can downregulate PTEN gene expression in cells transformed by anti-benzo(a)pyrene-trans-7,8-dihydrodiol-9,10-epoxide(Liu et al. 2010), indicating that miR-494 may be downregulated the expression of PTEN gene to promote the progression of gliomas. The current study showed that the expression of miR-494-3p in human glioma tissues and human glioma cell lines were higher than in normal brain tissues; miR-494-3p expression was inversely correlated with tumor grade.

PTEN is the first found in a double specific phosphatase activity of tumor suppressor genes, and it is closely associated with tumorigenesis after the p53 gene. PTEN protein plays an important role in cell growth, apoptosis, adhesion, migration, and infiltration. Some researchers have found that the PTEN gene has the most important role in the progression of gliomas (Li et al. 1997; Watanabe et al. 1998). PTEN loss can be activated AKT, thus promoting the progression of tumor. The expression of phosphorylated-AKT is the key factor representing the activities of PTEN/AKT pathways (Davies et al. 1998a, b; Sakata et al. 2002). The present study has found that miR-494-3p may target the gene and inhibit the expression of PTEN in mRNA and protein. Both in the glioma cell lines U251 and U87, miR-494-3p inhibitor can enhance the expression of PTEN to further suppress activated-AKT signaling and reduce the ability of invasion and proliferation in glioma cell lines. In addition, the expressions of BCL-2, Caspase-3, Caspase-9, and PTEN/AKT were changed by miR-494-3p inhibitor in U251 and U87 cell lines. These data have showed that the effect of miR-494-3p in the progression of glioma may be activated the signaling pathway of PTEN/AKT.

Most current therapies for glioma treatment are ineffective. The therapies for malignant gliomas need to seek novel targets and involve more effective and less toxic therapeutic strategies. PTEN gene is a tumor prognosis evaluation index and helps to study tumor action mechanisms; hence, gene therapy is a great significance to the diagnosis of tumor (Mueller et al. 2012). PTEN can prevent uncontrolled cell growth and inhibit tumor formation (Rasheed et al. 1997). Recently, with the increased research on miRNA, it could be confirmed by bioinformatics that miR-494-3p can reduce the expression of PTEN. Hence, it could be believed that miR-494-3p may be used as a molecular target of tumor treatment.

## Conclusions

The present study has found that the expression of miR-494-3p in glioma tissues was higher than in human normal brain tissues. miR-494-3p could act as a cancer gene to regulate the growth and invasion in vitro and in vivo in glioma. miR-494-3p inhibitor can reduce invasion, proliferation, and promote apoptosis in glioma cells. Thus, the miR-494-3p inhibitor might interrupt the activity of AKT pathways, independently of PTEN status. The miR-494-3p inhibitor could enhance the expression of PTEN and may act as an effective therapeutic strategy for suppressing the growth of glioblastoma.
